# Report of a Case of Fungosus Toxicum Paralyticus1Read before the Illinois Veterinary Medical and Surgical Association, January 14, 15, 1903.

**Published:** 1903-02

**Authors:** W. A. Swain

**Affiliations:** Mt. Pulaski, Ill.


### REPORT OF A CASE OF FUNGOSUS TOXICUM PARALYTICUS.[1]

By W. A. Swain, V. S.,
MT. PULASKI, ILL.

On the night of August 15, 1902, I was called to attend a valuable imported German coach stallion belonging to a company of men in Mt. Pulaski, Ill.

Upon my arrival I found the horse apparently suffering from a mild attack of indigestion, having a normal temperature and heart action. He did not seem to be suffering much, and I anticipated no serious trouble in soon having him all right.

I gave him a dose of ordinary colic mixture, such as I use for colic, and waited an hour, to find him with no material change. I then repeated the dose which I had given at first, and at the end of one hour there was still no perceptible change.

I now decided to administer an active physic, which I did, giving him four drachms of aloin, together with about four drachms of powdered zingiber. This seemed to afford him some relief. In about one-half to three-quarters of an hour from the time of administering the above physic the patient ceased manifesting uneasiness and stood quietly in his stall. His temperature at this time was normal; pulse, about normal; breathing, if anything, a shade slower than normal. He seemed to be rather stupid and sleepy, and paid little attention to what was going on around him.

I did not like his appearance, and, after consulting with the owners of the horse, we decided to call my father, Dr. S. H. Swain, of Decatur, in consultation. He was to come over on the early morning train. I then lay down and slept until a few minutes before he arrived, and did not see the horse again until he came, at which time we were surprised to find him manifesting strong symptoms of food poisoning. The first and one of the most prominent symptoms to attract attention at that time in this case was an apparent attempt to eat. The patient would thrust his nose into the hay, and to a casual observer would seem to be eating, but on closer observation it was seen that he was only nosing in the food in an unconscious

[1] Read before the Illinois Veterinary Medical and Surgical Association, January 14, 15, 1903.

condition. When led from his stall he was almost unable to stand, showing great lack of co-ordination, with pupils of eyes dilated and tongue hanging from mouth. The power of deglutition was entirely lost, making it impossible to administer medicinal agents per os. Temperature at this time, $98\frac{2}{5}°$; pulse, about 22; respiration very slow and stertorous.

On questioning the attendant we learned that two or three days prior to the manifestation of the above symptoms the horse had torn up the floor of his manger, which had a false or double bottom, and had eaten a quantity of mouldy chaff which had there accumulated. We pronounced it a case of fungosus toxicum paralyticus, and ascribed the cause to the eating of impure, mouldy food, as described above.

We gave him by hypodermic injection one-half grain strychnine and forty minims fluid extract digitalis, and had him moved about for about an hour. His temperature was again taken and found to have gone still lower, being now $96\frac{1}{2}°$, pulse almost imperceptible, respiration practically the same.

We tried two more hypodermic injections of strychnine and digitalis at intervals of an hour, with unsatisfactory results as to temperature and heart action. We then decided to try intravenous injections of stimulants; and for this purpose we agreed upon strong aqua ammonia, which was diluted with equal parts of distilled water and injected into the median subcutaneous vein, administering two-drachm doses every thirty minutes, with the most satisfactory results. Shortly after the first injection the temperature responded and rose to $98\frac{1}{2}°$, pulse 35, respiration more natural.

We administered in all four injections, thirty minutes apart, at the end of which time the temperature was 103°, pulse 48, and respiration practically normal. The pupils of the eyes had assumed their normal appearance, and the power of deglutition had returned, together with the use of the tongue, which was now kept within the mouth in a normal position.

The subsequent treatment consisted of stomachics: tr. gentian, two drachms; tr. zingiber, four drachms; tr. nux., two drachms; hydrochloric acid dil., one drachm, mixed and given at one dose, repeated three times daily for a period of about two weeks.

He was then changed to powders composed of about the above ingredients, less the hydrochloric acid, given three times daily in feed.

Our patient made a complete, although not very rapid, recovery, not being fully himself for about six weeks.

